# The use of initial dosing of gentamicin in the management of pyelonephritis/urosepsis: A retrospective study

**DOI:** 10.1371/journal.pone.0211094

**Published:** 2019-01-23

**Authors:** Silvia Ryanto, Mandy Wong, Petra Czarniak, Richard Parsons, Katherine Travers, Matthew Skinner, Bruce Sunderland

**Affiliations:** 1 School of Pharmacy, Faculty of Health Sciences, Curtin University, Perth, Western Australia, Australia; 2 Sir Charles Gairdner Hospital, Nedlands, Western Australia, Australia; Defense Threat Reduction Agency, UNITED STATES

## Abstract

**Objective:**

The primary objective was to determine if initial empirical intravenous dosing of gentamicin improved patient's outcomes in pyelonephritis/urosepsis compared with alternative IV antibiotic management.

**Design:**

Retrospective cross-sectional descriptive study.

**Setting:**

Public TertiaryTeaching Hospital serving adults in an urban centre.

**Participants:**

All adult patient records with a recorded diagnosis of any of pyelonephritis/urosepsis, urinary tract infection, UTI, complicated urinary tract infection, bacteriuria, symptomatic bacteriuria and asymptomatic bacteriuria from 2^nd^ February 2012 to 10^th^ May 2014 were reviewed. Only patients treated with an empirical regimen of one or more IV antibiotics were included in the study.

**Main outcomes:**

The primary outcomes were: duration of IV antibiotic treatment, time to resolution of symptoms and length of hospital stay (LOS). Secondary end points were: compliance with Therapeutic Guidelines: Antibiotic (electronic version) (eTG) for severe pyelonephritis/urosepsis and appropriateness of gentamicin use.

**Data analysis:**

Univariate and multivariable associations between baseline and demographic variables and the main outcomes were performed using Chi-square tests and a General Linear Model using the SAS version 9.2 software.

**Results:**

Of 295 patients reviewed 152 were prescribed one or more IV antibiotics and included in the study. Approximately half of the patients (n = 73, 48%) were prescribed IV piperacillin/tazobactam (Tazocin), while gentamicin was prescribed for 66 patients (43.4%). Of the 152 patients evaluated, 49 (32%) were given gentamicin first. Multivariable regression analysis showed that duration of IV treatment was shorter for those aged over 70 (40.2 hours vs 85.5 hours for those aged up to 70; p = 0.0074), and those who received gentamicin as first line treatment (41.3 hours vs 89.8 hours for those not receiving any gentamicin; p = 0.0312). After adjustment for age and gentamicin administration, there appeared to be no significant difference in duration of IV treatment for any other independent variables. No significant associations between the independent variables (gentamicin, age, gender, comorbidities, and eTG compliance) and either time to resolution of symptoms (median: 68 hours) or hospital LOS (median: 5 days) were observed.

**Conclusions:**

Neither time to resolution of abnormal symptoms nor length of stay were significantly influenced by an initial dose of gentamicin when the data were subjected to multivariable analysis. The age of the patient and pattern of gentamicin treatment were the dominant factors associated with duration of IV antibiotics. Piperacillin/tazobactam is not recommended in treatment guidelines for pyelonephritis/urosepsis but was the most commonly prescribed IV antibiotic. This requires review by the appropriate hospital clinicians.

## Introduction

Urinary tract infections (UTIs) are common in the community and hospital settings [1, 2].

Approximately 0.7% of community presentations are for UTIs and they are the fourth most common infection with a prevalence of 12.9%. Infection of the lower urinary tract is classified as simple cystitis [3]; but with kidney involvement pyelonephritis [4]. Uncomplicated cystitis more commonly occurs in young and sexually active women [5–7]. Pathogens from simple cystitis may invade the systemic circulation, which can progress to pyelonephritis/urosepsis [8]. The risk of this progression is increased by the presence of comorbidities including metabolic disorders (such as diabetes), functional or structural abnormalities (such as infected cysts, calculi, renal/bladder abscesses or renal stones) or unusual pathogens (such as yeasts) [9]. The most common pathogen isolated is *Escherichia coli* (*E*. *coli*), accounting for 70–95% of all UTI cases [10, 11].

Diagnosis of a UTI can be difficult in the elderly as distinguishing between asymptomatic bacteriuria and symptomatic infection especially when communication is poor may cause delays and therefore increase severity of the disease.

Sepsis is an inflammatory response to a systemic infection [12, 13], which can lead to multiple organ failure. This is a serious condition, with a high mortality risk [13–15]. Severe urosepsis accounts for approximately 5–7% of all sepsis cases, [16, 17] and has a 20–42% mortality rate [18]. As disease progression can occur quickly, immediate (within the first hour) IV antibiotic therapy can improve patient outcomes [19, 20].

Use of aminoglycosides has shown marked improvement in patient outcomes for infections involving some common Gram-negative organisms such as *E*. *coli*, *Klebsiella pneumoniae* and *Pseudomonas aeruginosa* [21, 22]. In Australia, the Therapeutic Guidelines: Antibiotic (electronic version) (eTG) [23], recommend gentamicin IV 4–6 mg/kg as a single dose with amoxicillin/ampicillin IV 2 g 6 hourly as first line empirical therapy in urosepsis [24, 25].

It was hypothesized that the initial administration of one or two IV doses of gentamicin as part of empirical therapy for severe pyelonephritis/urosepsis would result in improved patient outcomes. Therefore, the primary objective of this study was to determine whether patients receiving gentamicin in addition to other IV antibiotics showed improved outcomes compared with those receiving IV antibiotics in the absence of gentamicin. In addition the study aimed to determine the level of appropriate use of gentamicin in the selected population, with respect to standard treatment guidelines.

## Methods

### Design

A retrospective, cross-sectional descriptive study was conducted involving patients admitted for more than 24 hours to the Medical Assessment Unit (MAU) at a large 600 bed tertiary teaching hospital in Perth Western Australia, between February 2, 2012, and May 10, 2014. Staff at the hospital compiled a list of adult patients (male or female) whose diagnoses included any of the following: pyelonephritis/urosepsis, urinary tract infection (UTI), complicated urinary tract infection/UTI, bacteriuria, symptomatic bacteriuria and asymptomatic bacteriuria. The initial diagnosis would have usually been made by hospital emergency doctors. The ICD 10 codes were searched electronically and medical records of these patients were made available to researchers SR and MW. The study was approved by The Human Research Ethics Committee at Curtin University (Protocol Approval PH-12-14) and the Sir Charles Gairdner Hospital Ethics Committee with Quality Improvement (QI) Number 4559.

### Patients

Adult male and female patients initially treated with an empirical regimen of one or more IV antibiotics for the above diagnoses were included in the study. Patients who were given only oral antibiotics or no antibiotics were excluded. Data were collected from patient hospital records and included patient demographics (age, gender, weight, co-morbidities and whether the patient was a nursing home resident), penicillin allergies, primary and secondary diagnosis, symptoms on admission, and baseline signs (temperature, blood pressure, pulse). Co-morbidities for pyelonephritis/urosepsis were defined as any risk factors that contributed to the development of UTI, such as diabetes, indwelling catheter use, recurrent UTI, or renal obstructions. The total number of IV antibiotics, total duration of treatment, time and dates of each antibiotic regimen initiated and ceased, dose of each antibiotic, details of gentamicin dosing if given (dose, times and date), discharge medication regimen (if given) were also obtained from the patient medical records. Blood or urine microorganism identification and antibiotic sensitivity data were obtained from the hospital database to identify the microorganism and antibiotic suitability. Additional blood tests which included data on serum creatinine, gentamicin plasma levels, white cell counts (WCC), and C-reactive protein (CRP) were also recorded.

### Outcomes

The primary outcome was the time for resolution of pyelonephritis/urosepsis. Associated measures for this outcome included the duration (hours) of IV antibiotic treatment, duration (hours) to infection resolution and the hospital length of stay (LOS) (days). Time to resolution was calculated from the medical record observational charts, based on the timing of the first physiological parameter (temperature, pulse and blood pressure) indicating that the patient had returned from ‘abnormal’ to ‘normal’. Laboratory resolutions were defined as a WCC normalised to 4–12 x 10^9^/L and CRP decline of 50% from peak.

Secondary end points were the level of adherence with the eTG for the management of severe pyelonephritis/urosepsis and the appropriateness of gentamicin use in this sample population. Adherence to standard treatment guidelines was evaluated based on recommendations in the eTG^23^, where first line treatment is stated (for the complete study period) as gentamicin IV 4–6 mg/kg given as an immediate single dose with amoxicillin/ampicillin IV 2 g given every 6 hours [24,25]. For patients hypersensitive to penicillin, gentamicin alone can be used for initial therapy. If gentamicin is contraindicated, the alternative is either IV ceftriaxone 1 g or IV cefotaxime 1 g every eight hours [23]. Where the patient’s weight was not recorded and gentamicin was given, it was not possible to calculate whether the dose adhered with the eTG, and this group was classified as non-adherent as the correct dose could not be determined.

### Statistical analysis

Descriptive statistics were used to summarise the main outcome variables according to patient demographic and treatment profile (gentamicin treatment group, age, gender, co-morbidities, eTG adherence [23]), the type of antibiotic prescribed and culture strains. Univariate comparisons of the gentamicin treatment groups with respect to demographic variables were conducted with Chi-square statistics. Univariate analyses to assess any relationships between each of the main outcome measures: the duration of IV antibiotic treatment (hours), hospital LOS (days) and time to resolution (hours), and the independent variables were performed. Gentamicin dosing was classified into 3 groups as follows: 1) no gentamicin was given at all, 2) gentamicin given first as empirical therapy, or 3) gentamicin given following other IV antibiotics. Because the main outcome measures were not normally distributed (skewed), they were subjected to a Box-Cox transformation to find the transformation that would bring the outcomes closest to a Normal distribution. The logarithmic transformation was found to be optimum, so the outcomes were log-transformed before analysis. A separate univariate general linear model (GLM) for each of the main outcome measures was used to assess the statistical significance of any differences between groups. Fisher’s Least Significant Difference (LSD) post-hoc analysis was conducted to obtain the pairwise comparisons of the different gentamicin groups for each outcome. Following univariate analysis, a multivariable analysis was performed for each outcome, also using a GLM. All independent variables were initially included in each model, then the least significant variable were dropped, one at a time, until all variables remaining in the model were significantly associated with the outcome. The exception to this is that the variable indicating the gentamicin group was forcibly included in the model, as this variable was of primary interest. Pairwise interaction terms between main effects which remained in the model were also assessed for significance. The resolution of symptoms on the basis of blood tests were compared between gentamicin treatment groups using the Chi-square test. All statistical analyses were conducted using the SAS version 9.2 software (SAS Institute Inc, Cary, NC, USA, 2008), and a p-value ≤0.05 was taken to indicate a statistically significant association in all tests.

## Results

### Patients

A total of 295 records of patients with a diagnosis of UTI were reviewed. Based on the inclusion criteria, 152 with a diagnosis of severe pyelonephritis/urosepsis were included in the study. The remaining 143 patients were excluded on the basis that their sole treatment for UTI was an oral antibiotic (n = 127); the UTI was not treated at all (n = 6); the patient had other concomitant infections (n = 9); or had been prescribed prophylactic antibiotics (n = 1). Patients were predominantly female (n = 97, 64%), and the majority were over 70 years of age (n = 80, 53%). In addition, 53 (35%) patients had existing co-morbidities.

### Antibiotics prescribed

Table 1 shows the range and frequency of all IV antibiotics prescribed. There was a total of 252 antibiotics prescribed for the 152 patients. The table shows the numbers of each antibiotic prescribed, along with the percentage of patients who received it (based on a denominator of 152). The mean total number of different IV antibiotics prescribed per patient was 1.7. Piperacillin/tazobactam (Tazocin), was the most commonly prescribed antibiotic (given to 48% of patients), followed by gentamicin (43% of patients).

**Table 1:** Summary of all IV antibiotics prescribed.

IV Antibiotics Prescribed	Frequency of prescribing n = 152 n (%)
Piperacillin/tazobactam	73 (48.0)
Gentamicin	66 (43.4)
Amoxicillin	29 (19.1)
Ceftriaxone	29 (19.1)
Cefazolin	27 (17.8)
Ertapenem	7 (4.6)
Meropenem	7 (4.6)
Metronidazole	6 (3.9)
Cefepime	5 (3.3)
Cefotaxime	1 (0.7)
Azithromycin	1 (0.7)
Moxifloxacin	1 (0.7)

### Microbiology

The main microbiological cultures available from blood or urine samples are summarised in Table 2. It was found that *Escherichia coli* (n = 65, 42.5%) was the most commonly isolated pathogen, followed by *Klebsiella pneumoniae* and *Pseudomonas aeruginosa* (both n = 14, 9.2% respectively). Other culture strains included: *Citrobacter koseri*, *Proteus mirabilis*, *Klebsiella oxytoca*, *Serratia marcescens*, *Morganella morganii* and mixed growth.

**Table 2:** Summary of urinary/blood culture strains present in the 152 patient samples.

Culture	Frequency n (%)
*Escherichia coli*	65 (42.5)
*Klebsiella pneumoniae*	14 (9.2)
*Pseudomonas aeruginosa*	14 (9.2)
Others*	34 (22.4)
No growth detected	25 (16.3)

* *Citrobacter koseri*, *Proteus mirabilis*, *Klebsiella oxytoca*, *Serratia marcescens*, *Morganella morganii* and mixed growth

### Factors influencing patient outcomes

Review of the records showed that 31 (20%) adhered with guidelines. These included 11 (33%) that were prescribed ceftriaxone IV. There were 9 cases where gentamicin was given first and amoxicillin subsequently according to guidelines but the gentamicin dose could not be verified as the patient’s weight was not recorded. Table 3 shows the distribution of the independent variables (age, gender, comorbidities, eTG adherence across the gentamicin groups, with p-values to compare the profiles. The only significant association appeared to be with eTG adherence, where this was significantly higher amongst those who received gentamicin first (33%) than those who received it as a second line treatment (29%), or those who did not receive it at all (12%).

**Table 3:** Associations with gentamicin treatment group. Numbers in the table are the number of cases, and percentage of each gentamicin treatment group (column). P-values are calculated from the Chi-square statistic.

Variable	Gentamicin treatment group n (%)			p-value
	None	Second	First	
Age < = 70	43 (50)	10 (59)	27 (55)	0.7333
Gender: Male	30 (35)	6 (35)	19 (39)	0.8997
Comorbidities	31 (36)	5 (29)	17 (35)	0.8711
eTG compliant	10 (12)	5 (29)	16 (33)	0.0088

Table 4 shows the descriptive statistics for the duration of IV antibiotic (hours), time to resolution of symptoms (hours) and hospital LOS (days), within each of the groups defined by the independent variables. The univariate GLM identified significant differences in the duration of IV administration for the gentamicin group (p = 0.0248), for the age group (p = 0.0056), and for the eTG adherence variable (p = 0.0204). Pairwise differences for the gentamicin variable identified a significant difference in IV duration between patients given the drug first vs not at all (p = 0.0073), with those receiving the drug first having a significantly shorter duration of IV administration. Cases where the guidelines were followed also experienced a shorter duration of IV administration than those where guidelines were not followed (p = 0.0204).

**Table 4:** Univariate Statistics for the three main outcomes. Figures quoted are the median and interquartile range (IQR) from the 25^th^ to 75^th^ centile.

Variable	N (%)	Duration of IV (hours)	Time to Resolution (hours)	Length of Stay (days)
		Median (IQR)	Median (IQR)	Median (IQR)
Overall	152	28 (1–81)	68 (30–197)	5 (3–13)
Gentamicin				
None	86 (57)	46 (5–101)	83 (41–270)	5 (3–15)
Second	17 (11)	18 (2–51)	60 (30–97)	4 (2–6)
First	49 (32)	22 (1–48)	64 (16–149)	4 (2–12)
Age				
< = 70	72 (47)	45 (8–148)	73 (39–210)	5 (3–14)
71 or more	80 (53)	12 (1–57)	64 (25–166)	5 (2–13)
Gender				
Male	55 (36)	37 (1–94)	71 (39–233)	5 (3–16)
Female	97 (64)	28 (2–73)	67 (24–193)	4 (3–12)
Comorbidities				
No	99 (65)	32 (2–92)	73 (39–216)	5 (3–13)
Yes	53 (35)	17 (1–77)	58 (20–137)	5 (2–13)
eTG compliant				
No	121 (80)	37 (2–94)	73 (30–233)	5 (3–13)
Yes	31 (20)	4 (1–50)	65 (37–113)	5 (3–13)

While there appeared to be some differences in the time to resolution of symptoms, and hospital LOS (Table 4), the only variable which reached statistical significance was the gentamicin group and hospital LOS (p = 0.0496).

Multivariable analysis identified that only age group and gentamicin group were significantly associated with the duration of IV administration (Table 5). The variable indicating eTG adherence did not significantly contribute to this model, largely because eTG adherence and gentamicin group were strongly correlated. The GLM analyses were performed on the logarithm of the duration as there was considerable skewness in the raw measures. In addition to the results of the regression analysis (exponential of the regression coefficients in the column headed the ‘ratio’ and p-values), Table 5 also includes the adjusted means based on the original scale (hours) for ease of interpretation. The values in the ‘Ratio’ column show the ratio of the hours of each group relative to the reference group. While the adjusted means based on the raw data are not strictly accurate because of the skewness in the raw data, they give an indication of the means on the original scale which may aid the understanding of the data. The mean duration of IV treatment for those aged over 70 years was approximately 40 hours compared with 85 hours for those up to the age of 70 (confidence intervals and p-values are shown in Table 5). The duration of IV treatment for those who received gentamicin first was significantly lower than those who did not receive gentamicin at all, but not significantly different from those who received the drug second (based on the confidence intervals for the ratios).

**Table 5:** Multivariable analysis for the duration of IV antibiotics. P-values, the ratio and their confidence intervals were obtained from a multiple linear regression of the logarithm of the duration on age and gentamicin group. The adjusted mean hours are based on the raw (untransformed) data.

Variable	Ratio (hours)	95% CI for B	Adjusted Mean (hours) (95% CI*)	p-value
Age				0.0074
< = 70	1 (reference)		85.5 (53.7–117.3)	
71 or more	0.42	0.22–0.79	40.2 (10.7–69.8)	
Gentamicin group				0.0312
None	2.53	1.26–5.05	89.8 (64.3–115.4)	
Second	1.52	0.51–4.53	57.6 (0.03–115.2)	
First	1 (reference)		41.3 (7.4–75.2)	

* Confidence Interval

Table 6 shows the numbers and percentages of patients with a recorded resolved CRP and WCC upon discharge for each sub-category of gentamicin administration. The p-values (obtained from a Fisher’s Exact test because numbers in some cells were small) suggest that gentamicin administration was not associated with CRP and WCC resolution in this study. There also appeared to be no association between the pattern of gentamicin use and renal function.

**Table 6:** The association between pattern of gentamicin prescribing and laboratory data^a^.

Variable	Gentamicin group n (%)			p-value
	None	Second	First	P-value
CRP resolved				0.7952
No	34 (54)	5 (56)	21 (62)	
Yes	29 (46)	4 (44)	13 (38)	
WCC normal				0.7548
No	27 (35)	4 (31)	11 (27)	
Yes	51 (65)	9 (69)	29 (73)	
Renal clearance				0.7172
< = 60 L/min	61 (71)	11 (65)	32 (65)	
> 60 L/min	25 (29)	6 (35)	17 (35)	

^a^ Numbers in the table are the number of cases, and percentage of each gentamicin group. P-values were obtained from Fisher’s Exact test.

^b^ CRP = C-reactive protein; WCC = white cell count

## Discussion

This study has investigated empirical IV antibiotic treatment prescribed for 152 patients for severe pyelonephritis/urosepsis with one or more IV antibiotics. The overall duration of IV antibiotic treatment was significantly shorter for patients given gentamicin empirically as initial treatment compared to patients not given gentamicin at all. eTG adherence was a factor in reducing IV antibiotic duration but did not influence the other outcome parameters. Duration of IV therapy is an important indicator as patients usually need to cease IV antibiotics before discharge can be considered. The large spread of the data as can be seen from the wide ranges possibly limited other significant findings.

This result suggested that an initial dose of IV gentamicin positively affected patient outcomes in terms of IV antibiotic treatment length and hospital LOS. Gentamicin has long been the antibiotic of choice in the treatment of severe cases of suspected gram-negative sepsis due to its effectiveness against *Pseudomonas aeruginosa* [26, 27]. Some patients were infected with Ps*eudomonas aeruginosa*. However *E*. *coli* was the main pathogen, a finding which has previously been documented [10, 11]. A recent study reported 54% of *E*.*coli* strains found in urine were sensitive to gentamicin [28]. Adhering to eTG requirements also produced improved outcomes for duration of IV antibiotics, but this was correlated with the pattern of gentamicin administration which was the dominant factor. These data also included the initial administration of ceftriaxone.

A limitation in this study was the imprecise recording of the diagnosis. For this study, the initial prescribing of an IV antibiotic in the treatment regimen for “UTI” was considered an indication of severe pyelonephritis/urosepsis. Patients who were only given oral or no antibiotics, were excluded as this suggested a diagnosis of acute cystitis or mild pyelonephritis as opposed to severe pyelonephritis/urosepsis. The combination of the imprecise diagnosis and some missing data for various parameters contributed to some limitations in the scope of data analysis. In addition, the wide ranges of observed LOS, duration of treatment and time to resolution, combined with the small to moderate sample size mean that the study may be susceptible to Type II errors (true associations may have been missed).

Age was analysed against duration of IV antibiotic treatment, duration to disease resolution and hospital LOS. Notably the older group (> 70 years) had a significantly shorter duration of IV antibiotic treatment possibly owing to the awareness of increased risk of toxicity, such as nephrotoxicity [29], in the elderly population.

Adherence with eTG [23] recommendations was low with 31 (20%) of IV antibiotics prescribed in accordance with recommendations. This was partially caused by a lack of recording of patient weight which then did not permit verification of the dose of gentamicin and these were classified as non-adherent. Prescribing IV ceftriaxone would have also influenced the eTG adherence outcome. In several cases IV ceftriaxone was initially prescribed followed by IV gentamicin. This may have been once urinary excretion had been determined. A high number of empirical prescriptions for piperacillin/tazobactam was found, or gentamicin followed by piperacillin/tazobactam which are not treatment options under the guidelines. A study conducted by Khan *et al*.[30], noted the significant widespread and inappropriate use of this antibiotic in the hospital setting [30]. It is noted that piperacillin/tazobactam is not a restricted antibiotic at the study hospital. Hence such an antibiotic can be prescribed without permission even when not recommended in guidelines. However even when an antibiotic is effective against the pathogen detected, it does not mean it is the most appropriate.

The lack of adherence to the eTG [23] recommendations could be due to a lack of awareness of the current guidelines, uncertainty of differential diagnosis and complex co-morbidities [30] (such as renal failure that could contraindicate first line empirical gentamicin treatment as stated in the eTG [23]). However, it has been well documented that aminoglycosides, such as gentamicin, have consistently demonstrated bactericidal activity as well as a post antibiotic effect, hence they continue to manage both Gram-positive and Gram-negative bacterial growth after they have been ceased [15, 31, 32]. Lee et al. [33] also reported that guideline non-concordant empirical antibiotic treatment of acute pyelonephritis produced a lower early clinical response rate and increased hospital LOS.

A secondary aim of this study was to determine the appropriateness of the use of gentamicin in this population. Unfortunately, reliable conclusions could not be drawn, as crucial data, such as patients’ weight and renal function were inconsistently recorded. However, with the available data, a chi-squared analysis was conducted on renal clearance and gentamicin dosing. It was found that 25 out of 86 patients (29.1%) who were not given gentamicin at all had an eGFR > 60mL/min, and this was not significantly different from the clearance rate for those patients who were taking gentamicin (Table 6). Gentamicin also had no significant effect on either CRP or WCC resolution times. These findings could have been partially influenced by missing blood test results for a number of patients, in particular those admitted for two days or less. As a result, these patients were unable to be included in that analysis, limiting any conclusions that may be drawn. Although it could be possible that gentamicin was reserved for less sick patients (and hence shorter duration of treatment and stay in itself) the renal function data (influenced by age and illness) show no such correlation.

Further limitations of this study include the retrospective nature of the study (no control over the treatment allocated to each patient), which may affect comparability between groups. Some patient documentation was inconsistent and some important data were missing from some records. These included the patient weight (which is an important determinant of appropriateness of gentamicin dosing), renal function, and blood test results (in particular CRP and WCC). The study was conducted at a single hospital which may limit the generalisability of the findings. Often a general coded “UTI” diagnosis was recorded rather than specifically cystitis or pyelonephritis/urosepsis. This approach has been adopted since this study investigated initial empirical prescribing rather than when a final diagnosis was made. Scrutiny of patient data was necessary and 143 patients were rejected, to identify those initially prescribed IV antibiotics. It is therefore possible that some confounding with respect to indication could have arisen, but those patients were more likely to have been excluded rather than included.

## Conclusions

There were univariate associations indicating that an initial dose of gentamicin significantly improved patient outcomes in terms of duration of IV treatment, and this association persisted after adjustment for age.

Failure to follow guidelines may be due to a lack of awareness of the current eTG recommendations or caution in empirically prescribing gentamicin in the study population. As such, it would be beneficial in future to evaluate prescribers’ views regarding the current standard treatment guidelines. The role of piperacillin/tazobactam as initial IV antibiotic management in pyelonephritis/urosepsis requires review, especially in the light of these findings.
